# Response induced in *Mycoplasma gallisepticum* under heat shock might be relevant to infection process

**DOI:** 10.1038/s41598-017-09237-7

**Published:** 2017-09-12

**Authors:** Ivan Butenko, Anna Vanyushkina, Olga Pobeguts, Daria Matyushkina, Sergey Kovalchuk, Alexey Gorbachev, Nicolay Anikanov, Gleb Fisunov, Vadim Govorun

**Affiliations:** 1 Laboratory of Proteomic Analysis, Federal Research and Clinical Centre of Physical-Chemical Medicine, Moscow, 119435 Russia; 2 Laboratory of Proteomics, Shemyakin-Ovchinnikov Institute of Bioorganic Chemistry, Moscow, 117997 Russia; 3 Moscow Institute of Physics and Technology (State University), Dolgoprudny, 141700 Russia

## Abstract

Despite the fact the term “proteome” was proposed to characterize a set of proteins in one of mycoplasma species, proteome response to various exposures in this bacteria are still obscure. Commonly, authors studying proteomic response on perturbation models in mycoplasmas use single approach and do not confirm their findings by alternative methods. Consequently, the results of proteomic analysis should be validated by complementary techniques. In this study we utilized three complementary approaches (SWATH, MRM, 2D-DIGE) to assess response of *Mycoplasma gallisepticum* under heat stress on proteomic level and combined these findings with metabolic response and the results of transcriptional profiling. We divide response into two modes – one is directly related to heat stress and other is triggered during heat stress, but not directly relevant to it. The latter includes accumulation of ATP and shedding of antigens. Both of these phenomena may be relevant to evasion of host’s immune system and dissemination during mycoplasmosis *in vivo*.

## Introduction

More than 20 years have passed since the term “proteome” was first used to characterize cell protein composition. In the seminal study two-dimensional gel electrophoresis and MALDI-TOF mass spectrometry were used on *Mycoplasma genitalium* ^1^, the organism with the smallest known naturally occurring genome encoding minimal set of open reading frames that supports free genome replication and cell division in a synthetic medium. Despite numerous research efforts, key principles in regulation of the genome activity of Mycoplasmas are still obscure; they are characterized by an unexpected complexity coded by minimal genome context. Thus, the investigators have to return to the studies of mycoplasmas’ proteome architecture as their proteins are orchestrated to provide cell survival under wide range of optimal and non-optimal conditions. The cells of pathogenic mycoplasmas are characterized by high plasticity and adaptive potential to evade host immune surveillance, acquire antimicrobial resistance, and to disseminate to new hosts. However, adaptive responses of these cells are not associated with highly effective functioning of transcription regulators or other known mechanisms as the genes of these factors are not annotated in Mollicutes genomes.

As we reported previously, gene expression in mycoplasmas is mostly affected by heat stress^2^. Considering that mycoplasmas are parasites, since temperature rise is a standard host response to bacterial invasion, it is reasonable to assume that, in course of host-mediated evolution heat stress could have become an important regulation factor for mycoplasmas as it may serve as a universal marker of certain stages of host’s immune response.

*Mycoplasma gallisepticum*, an avian pathogen, is one of the well-studied members of Mollicute class, and phylogenetically it is closely related to human mycoplasmas *M*. *pneumoniae* and *M*. *genitalium*. Considering their compositional features, i.e., a reduced genome, small number of proteins and metabolic paucity, mycoplasmas are a common object for systemic analysis^3–9^.

Earlier we performed transcriptome analysis in *M*. *gallisepticum* and demonstrated the role of promoter structures and transcript 3′-ends in transcription regulation under heat stress^2^. The study of *M*. *gallisepticum* transcriptional activity parameters and the structure of its promoter and terminal regions allowed us to formulate the hypothesis on the regulation of transcriptional activity without involving annotated transcriptional factors (“regulation without regulation”), i.e., by means of structural features of promoters and terminal sequences. We also revealed weak correlation between variable transcriptional activity of *M*. *gallisepticum* and response for proteins encoded by variating transcripts. Further, we determined the sequences of transcripts interacting with ribosomes during the exposure to elevated temperature on *M*. *gallisepticum* cells and revealed certain interaction patterns between mRNA and ribosomes in response to heat stress^10, 11^.

As the next step, in this paper, we made an effort to correctly determine specific changes in cell protein and metabolite levels during thermal adaptation. We described several components comprising relevant response to heat stress, and accompanying ones that may be involved in the development of pathogenic process. We revealed response features at protein and metabolite levels and compared them with data on transcription-level response to heat stress in *M*. *gallisepticum*.

To analyze the changes in protein abundance of *M*. *gallisepticum*, we used SWATH (Sequential Window Acquisition of all Theoretical Mass Spectra) technique - high-performance data-independent label-free method of LC/MS acquisition. We also complemented LC/MS data with the results of classic two-dimensional gel electrophoresis with differential staining using cyanine dyes. Additional validation was performed with label-free MRM (Multiple Reaction Monitoring) method. In addition, we identified 100 metabolites and determined the changes in their levels under heat stress using LC/MS. Using transcriptional profiling data^2^, we performed integrative analysis including mRNA, protein, and metabolite-level response to heat shock. Our major findings are 7-fold increase in ATP, acceleration of cell metabolism with the transition to oxidative stress due to the increase in peroxide production rate, and antigen “release”.

## Results

### Complementary proteomic approaches provide exhaustive description of Mycoplasma gallisepticum status under heat shock

**Figure 1:**
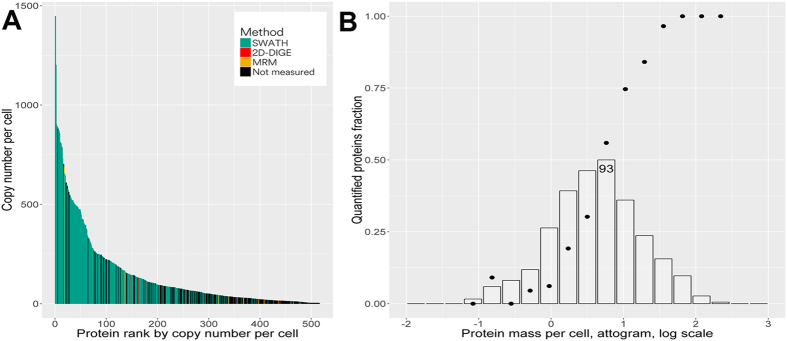
Coverage of *Mycoplasma gallisepticum* proteome by three complementary quantitative approaches. (**A**) Protein copy number spans across 4 orders of magnitude. Large-scale quantitative effort is heavily biased towards most abundant proteins. (**B**) Bars - estimated protein distribution by mass per cell; Points - fraction of proteins successfully quantified.

Shotgun LC/MS analysis revealed 9379 unique tryptic peptides for 588 of 836 known protein-coding ORFs (on average, 15 peptides per protein) that were used to create spectral library. SWATH-based quantitative analysis met reproducibility criteria for 237 proteins (Supplementary Table S2). Absolute concentrations (in arbitrary units) were determined by iBAQ (Intensity-Based Absolute Quantification) method and reduced to the number of protein copies per cell using the data for related *M*. *pneumoniae* ^3^ for 516 proteins. Copy number ranged from 1 to ~1500 protein copies per cell (Fig. 1A and Supplementary Table S2).

237 proteins that were successfully quantified by SWATH made up 40% of identified proteins and were estimated to comprise 82% of proteome by mass. Also, among the proteins with over 100 copies per cell, 79% (i.e., 155 of 196) were quantified (Supplementary Table S2). Remaining proteins were either short (up to 200 amino acid residues, for example, ribosomal proteins) or membrane proteins and proteins with highly homologous sequences that could not be distinguished correctly. Even though iBAQ predicted high abundance of these proteins, some features of their sequences (extensive hydrophobic or non-unique regions of the sequence, short length) did not allow to choose reproducibly observed unique peptides for them (Fig. 1).

SWATH LC/MS experiment was performed on 6 independent biological replicates and each sample was analyzed in triplicate. Every independent experiment was performed on cultures sampled at logarithmic growth phase just before thermal exposure, after 30 min incubation at 46 °C, and after additional incubation for 2 hours at 37 °C. It was shown earlier that 46 °C is the highest temperature, under which most cells remain viable (sublethal conditions)^12^ as was determined by number of colony forming units that are formed by diluted culture after subjecting it to stress conditions of different power. Standard error across 6 biological repeats was less than 0.18 in 80% of measurements.

**Figure 2:**
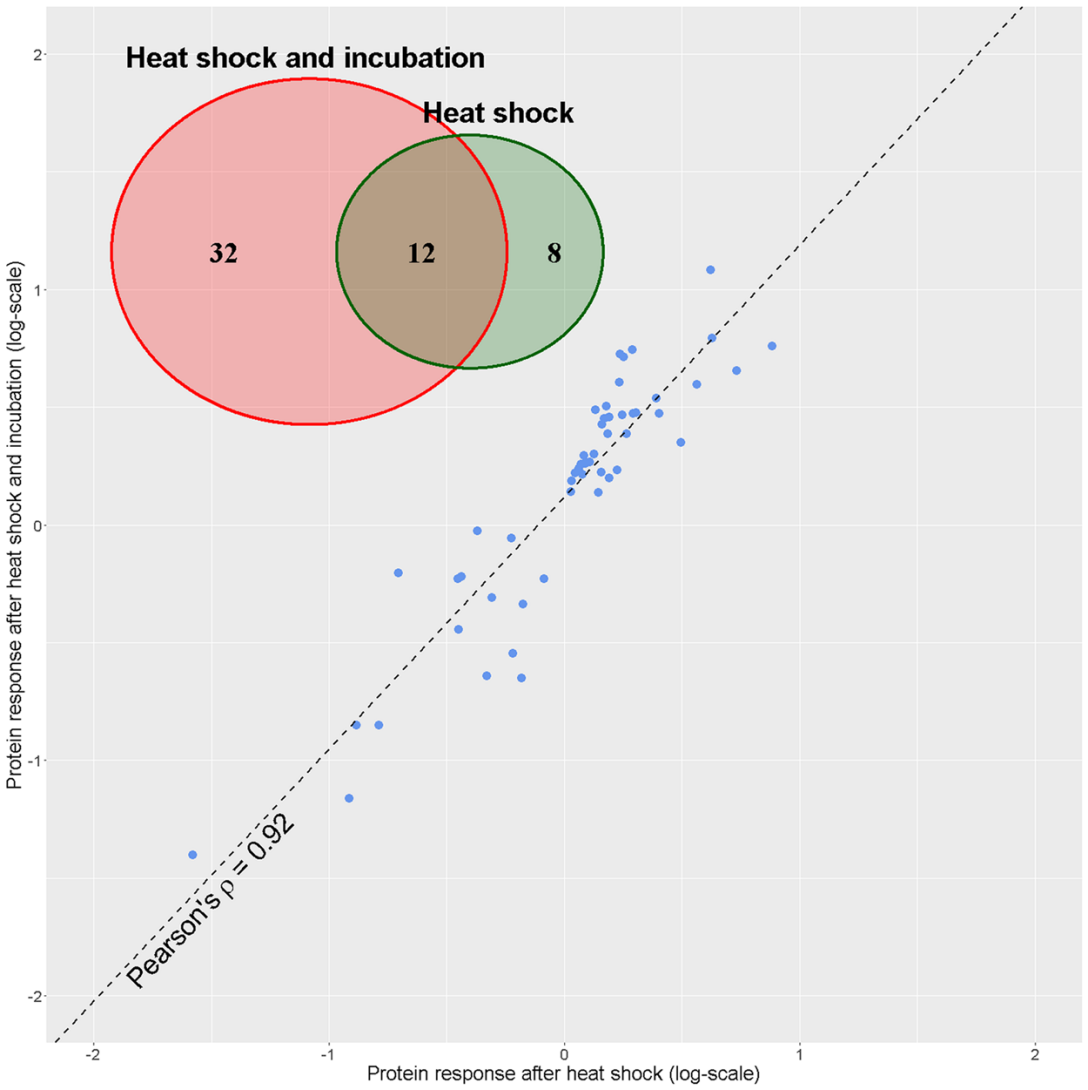
Differences in the abundance of significantly changing proteins immediately after heat shock and additional incubation. Statistical significance of changes in protein abundance results in barely overlapping lists of response participants, but strong correlation between relative changes in proteome after heat shock and additional incubation supports their combination.

Significant differences were shown for 52 proteins (Supplementary Table S1) (20 proteins were differently represented between the control culture and the culture immediately after heat shock and 44 proteins - between the control culture and the heat shock culture after additional incubation, all with p-value below 0.05). Pearson’s correlation coefficient between relative abundance changes 30 min after heat shock and 120 min after additional incubation was 0.92 for these 52 proteins (Fig. 2).

**Figure 3:**
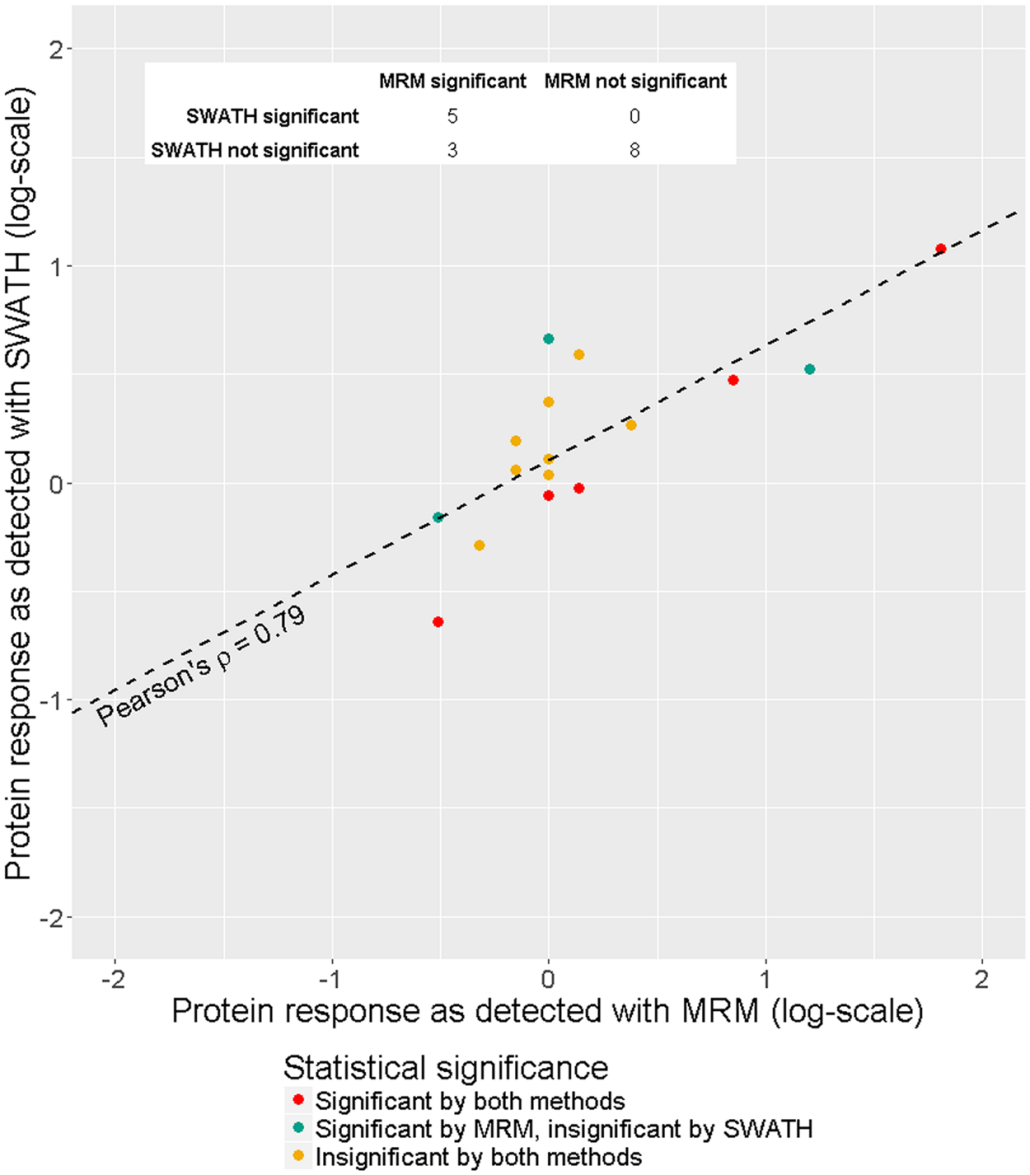
Large-scale quantitation with SWATH compared with targeted MRM measurements. Big scope of SWATH technique comes at cost of precision, but not accuracy, so that higher number of replicates is required to reach the same significance levels.

To validate the data obtained using SWATH technique, we repeated analysis with common MRM method, where 16 proteins whose abundance was 5–110% higher or smaller after heat shock compared with control culture and 14 additional proteins were targeted. 5 of 16 proteins measured in both SWATH and MRM experiments demonstrated significant differences in both with Pearson correlation coefficient of 0.99 for log-transformed fold changes (Fig. 3). Although, for 3 proteins SWATH experiment failed to reach significance threshold, overall correlation for 16 proteins reached 0.79.

**Figure 4:**
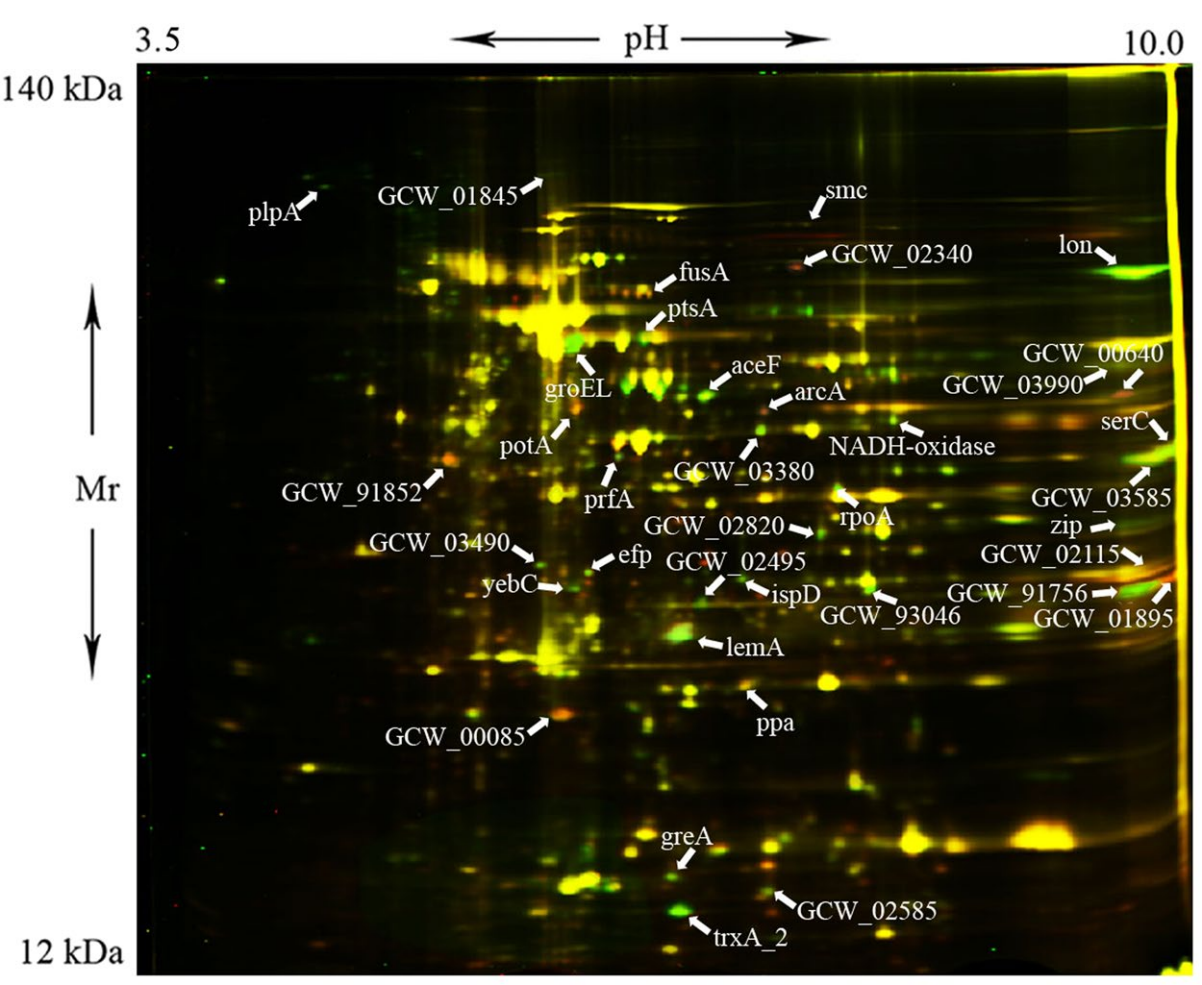
2D DIGE analysis of *M*. *gallisepticum* after heat stress (Cy3, green) and the laboratory strain *M*. *gallisepticum S6* (Cy5, red). Under Heat stress conditions *M*. *gallisepticum* was cultivated at 46 °C for 30 minutes.

Since less than a half of identified proteins were available for extensive LC/MS analysis, complementary proteomic analysis was also performed to possibly extend the proteome coverage, i.e., findings made with bottom-up proteomics were supplemented with the results of classic two-dimensional gel electrophoresis and differential staining with cyanine dyes (2D-DIGE) (Fig. 4). Values based on the ratio of the fluorescence intensity for the channels Cy3/Cy5 were counted using the PDQuest software package (Bio-Rad). Mycoplasma under heat shock marked by green Fluorescent dye (Cy3), a control laboratory strain of *M*. *gallisepticum S6* - red (Cy5). This approach revealed 36 additional proteins involved in heat shock response that were not shown as differing significantly in neither SWATH nor MRM experiments (Supplementary Table S1).

Therefore, a total of 96 proteins were identified (Supplementary Table S1) whose abundance was significantly different in the cultures exposed to heat stress. Quantitative data on 60 proteins was obtained by LC/MS thus providing the assessment of relative abundance changes. Only 4 proteins (VlhA.2.01, one of the major Vlh proteins; OsmC, osmotic protector; RpsG, ribosomal protein; and chaperone ClpB) exhibited increase or drop in abundances by factor of 2 or higher, while the changes for 35 proteins lied in range from 1.1 to 1.4.

Thus, at protein level *M*. *gallisepticum* response to heat stress has several key components (Supplementary Table S1). First, the abundance of major chaperones (DnaK, ClpB, GrpE, GroEL, and DnaJ), protectors against oxidative stress (OsmC and TrxA, thioredoxin) and proteins involved in DNA repair^12^ (NrnA and Nfo) increases. Second, the level of major variable lipoproteins (Vlh.A.1.01, Vlh.A.2.01, and Vlh.A.3.03) reduces. Abundance of proteins involved in energy metabolism (glycolytic proteins, ionic transport proteins) increased. In addition, ribosome undergoes significant re-modelling which involves changes in the stoichiometry of structural ribosome proteins (RplA, RplW, RpmC, RpmF, RpsG, RpsH and RpsM, or L1, L23, L29, L32, S7, S8 and S13) and transcription elongation factors P and G (Efp and FusA). Moreover, the expression of transcriptional factor GreA increases immediately after heat shock while the abundance of RNA polymerase β- and β‘-subunits (RpoB and RpoC) decreases and the abundance of RNA-degradosome protein (RNaseJ) increases after the incubation.

### Relevant protein response arises with little support on transcription level

Majority of changes in cell proteome in response to heat stress is relatively small and activity of affected proteins is either directly required to maintain homeostasis under elevated temperature or presumably required to mediate proteome regulation itself (Supplementary Table S1). However, in our previous works we observed changes in RNA profile that were much more significant but lacked such specificity^2, 11^.

Due to lower limit of quantitation for transcription profiling techniques in terms of copy number per cell when compared to proteomic methods, quantitative data on the changes in protein abundance were obtained only for 25% of transcripts (106 of 431) whose abundance changed by factor of 2 or more and reached q-value below 0.05. However, even when both mRNA and protein were successfully quantified, gene expression was poorly correlated with protein abundance.

**Figure 5:**
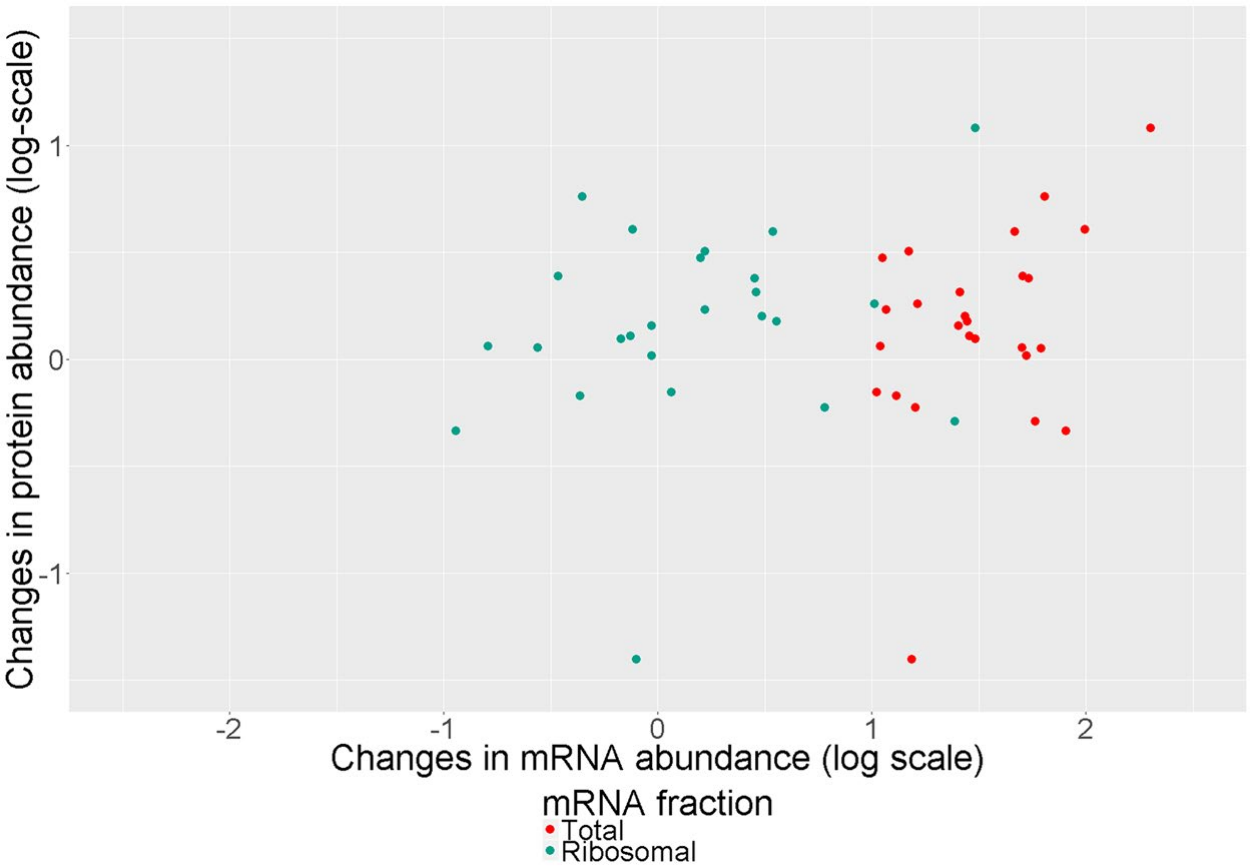
Correlation between mRNA and protein responses in *M*. *gallisepticum* under heat stress.

In fact, Pearson correlation coefficient between the number of RPKM (Reads Per Kilobase Million, proportional to mRNA copy number per cell^11^) and protein copy number was equal to 0.69 for 516 identified proteins in case of *M*. *gallisepticum* cultivated under standard conditions (control case), which is close to values obtained for other organisms^13^. At the same time, after heat shock Pearson correlation coefficient between log-transformed fold changes of mRNAs and corresponding proteins reached 0.32 for 26 proteins and transcripts for which mRNA abundance increased by factor of 2 or more in total mRNA fraction and corresponding q-value fell below significance threshold of 0.05 (Fig. 5). On the contrary, for all transcripts whose abundance reduced by factor of 2 or more, the same coefficient did not exceed 0.08 in both conditions considered in proteome experiment. Changes in mRNA abundance in ribosome-bound fraction in the same test has shown negative correlation coefficient as low as 0.06 immediately after heat shock and −0.1 after additional incubation.

While certain correlation was observed, 75 quantified proteins did not reveal any significant changes although corresponding mRNAs’ abundance drops or increases by factor of 2 or higher, but even when trends for mRNA and protein are the same, the effect on mRNA level is on average 2 times more intense. These findings indicate that proteomic response is regulated not only by the changes in RNA abundance but also by other factors, which may include the kinetics of interaction between mRNA and ribosomes (i.e. ribosome queuing).

### Nucleoside and terpenoid biosynthesis are the most affected branches of Mycoplasma gallisepticum metabolome under heat stress

Previously, we identified over 100 of 225 *M*. *gallisepticum* intracellular metabolites by LC/MS^14^. Among metabolites detected were components of all major metabolic pathways, i.e., glycolysis, metabolism of amino acids, purines, and pyrimidines, terpenoid biosynthesis. Metabolic map of *M*. *gallisepticum* was reconstructed using metabolome and proteogenomic analysis data (Supplementary Figure S1).

In this study, we analyzed changes in metabolite content under heat shock. We revealed several major trends in *M*. *gallisepticum* metabolome changes under heat shock. Most metabolites whose concentration increased under heat stress were components of purine and pyrimidine metabolism (Supplementary Table S3). We demonstrated an increase in four major nucleosides as well as xanthosine and deoxyguanosine levels and major nitrogenous base (cytosine, uracil, guanine, adenosine, and xanthine) abundances. Despite increased xanthine concentration, we failed to detect increased levels of inosine or its metabolites. We also detected CMP and GMP accumulation and reduced AMP abundance (Supplementary Table S3).

**Figure 6:**
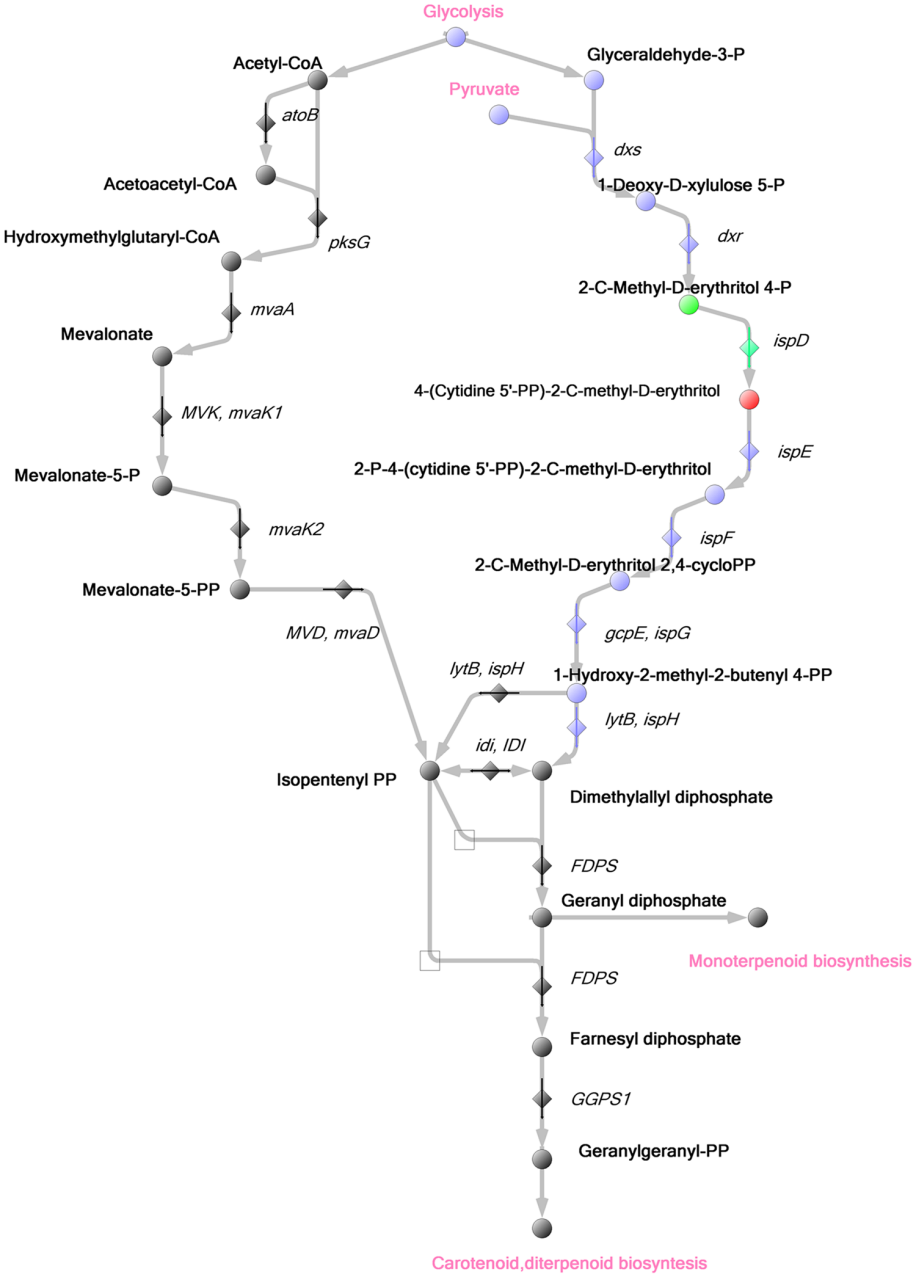
Terpenoid biosynthesis in *M*. *gallisepticum*. Biochemical reactions of terpenoids biosynthesis annotated for *M*. *gallisepticum S6* indicated in violet. Metabolites involved in this pathway are shown as circles; enzymes are shown as diamonds; Metabolites and proteins that demonstrated increased level under heat shock marked in green, decreased level – in red.

We revealed accumulation of terpenoid biosynthesis metabolite, 2-C-Methyl-D-erythritol-4-phosphate, in cells exposed to heat shock while the abundance of another metabolite, 4-(Cytidine-5′-diphospho)-2-C-methyl-D-erythritol, significantly decreased (Supplementary Table S3). Figure 6 illustrates terpenoid biosynthesis in *M*. *gallisepticum* based on genomic data. 4-(Cytidine-5′-diphospho)-2-C-methyl-D-erythritol (decreases under heat shock) is synthesized from 2-C-Methyl-D-erythritol-4-phosphate (increases under heat shock); this reaction involves CTP and is catalyzed by IspD (2-C-methyl-D-erythritol 4-phosphate cytidylyltransferase).

The levels of major osmotic protectors, amino acids valine, proline, and glutamate, reduce under heat stress (Supplementary Table S3). These data suggest decreased intracellular osmotic pressure. As some amino acids are known to exhibit properties of chemical^15^ chaperones, decrease in their abundance during heat stress could have been compensated with action of major chaperones DnaK, DnaJ and ClpB.

### ATP level increases considerably under heat stress

**Figure 7:**
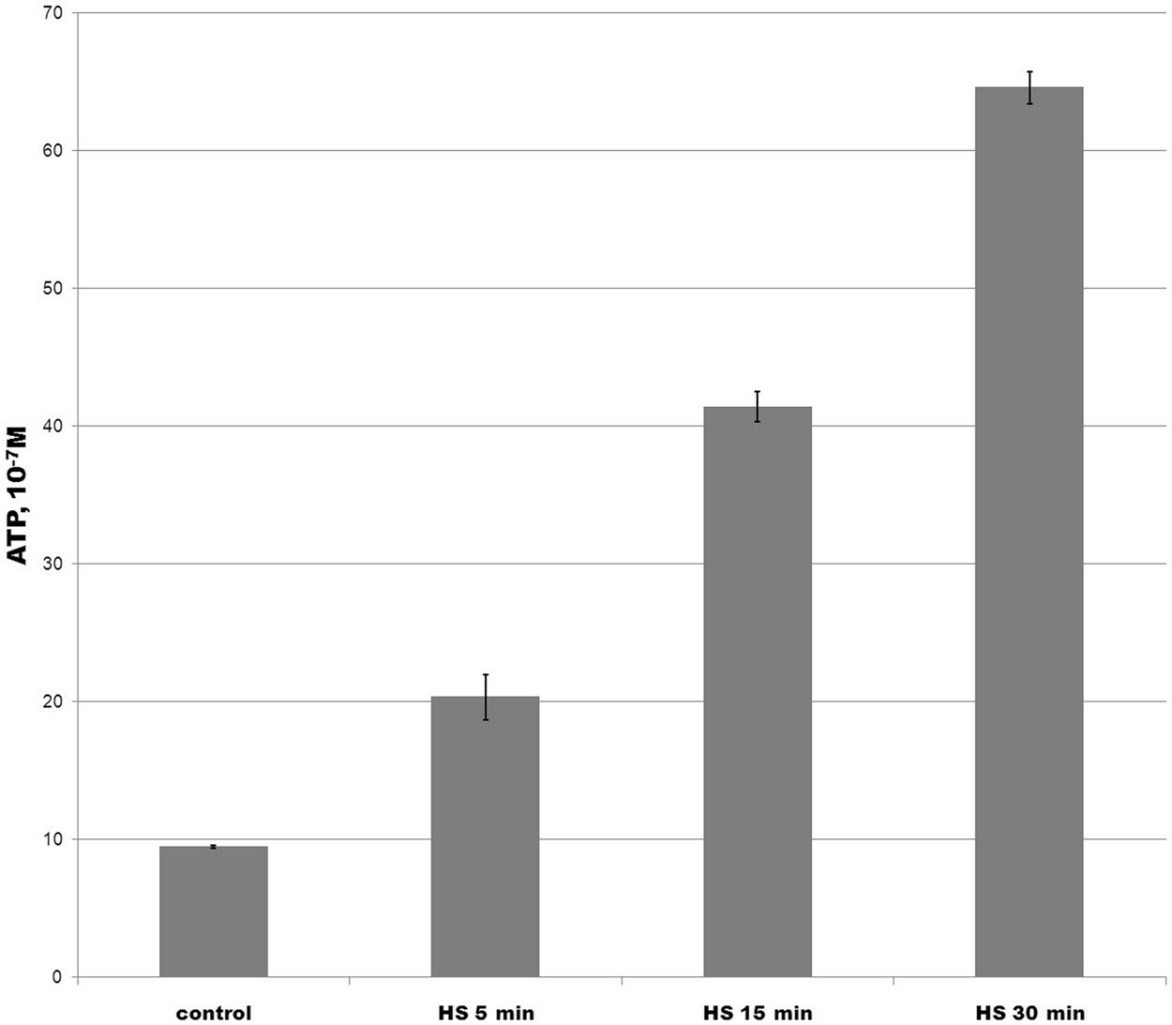
Measurement of the ATP concentration under heat stress. A sterile medium for *M*. *gallisepticum* culturing was used as baseline. The data represent the mean (±SD) of three independent experiments performed in triplicate.

We measured the level of intracellular ATP using bioluminescence system based on luciferin-luciferase reaction and revealed a 7-fold ATP increase under heat stress. Cultures exposed to heat stress in log-phase (5, 15, or 30 min at 46 °C) demonstrated increased intracellular ATP levels as compared with log-phase culture grown in normal conditions at 37 °C. The experiment was performed as three independent biological repeats with three technical replicates each (Fig. 7).

### Continuous heat shock promotes reduction of hemagglutination

**Figure 8:**
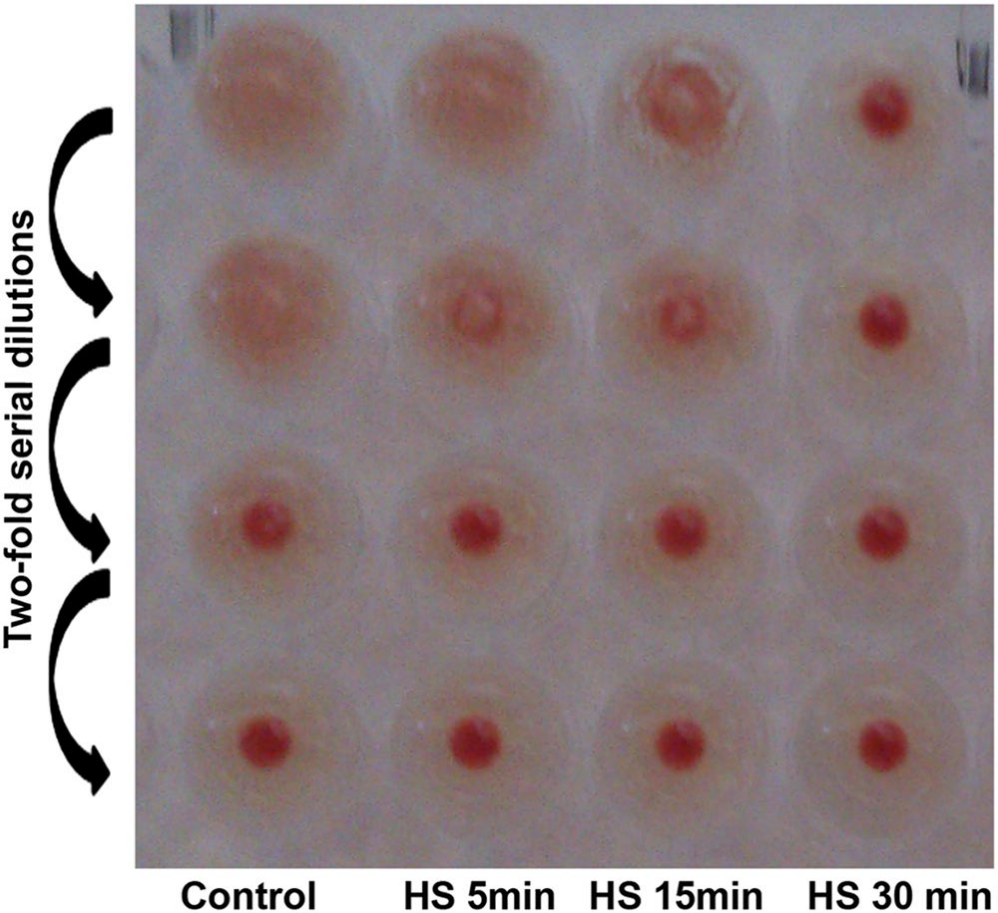
Hemagglutination test in *M*. *gallisepticum* after heat stress. Two-fold dilutions of *M*. *gallisepticum* cells are represented top-down. The conditions are on the horizontal axis, left-to-right: control and heat stress 5 min, 15 min, and 30 min. Hemagglutination decreased at least 4-fold after heat stress.

The results of proteomic profiling demonstrate decreased abundance of major superficial Vlh antigens (Vlh 2.01, VlhA.1.01, VlhA.3.03) (Supplementary Table S1) in *M*. *gallisepticum*. The loss of superficial antigens responsible for hemagglutination should result in significant inhibition of this process. We performed hemagglutination test with chicken erythrocytes. Negative results form a compact spot in the center of round-bottomed plates. Positive results form a uniform reddish color across the well. Inhibition of hemagglutination was observed as early as 5 min after heat shock. After 30-min heat shock, hemagglutination reduced 4-fold (Fig. 8).

## Discussion

To describe the process of *M*. *gallisepticum* adaptation to heat stress in details, we measured the changes in protein and metabolite levels. Since it was shown earlier^2^ that protein composition changes are relatively small, we used a combination of three complementary methods of quantitative proteomic analysis, i.e., extensive SWATH bottom-up analysis, targeted MRM bottom-up analysis, and two-dimensional gel electrophoresis with differential staining using cyanine dyes (top-down analysis). Among the proteins whose levels increase under heat stress, well-known heat shock proteins, chaperone ClpB and co-chaperonin GroEL, are of special importance. Data on increased abundance of these proteins agree with earlier studies on heat shock response in many bacteria including *Mycoplasma* species^16–18^.

Currently, weak correlation between mRNA and protein is described for a variety of bacteria^19–22^. In *E*. *coli*, mRNA level changes could explain variable protein abundance to a more considerable degree as compared with other bacteria but no more than by 50%^22^. Nevertheless, bacterial cells require determinate regulatory system to successfully adapt to environment perturbations. In *M*. *gallisepticum* and other Mollicutes, total genome reduction was accompanied by the loss of transcription regulators^11^. Therefore, a more important role of post-transcription regulation can be hypothesized.

In this study, we observed large-scale changes in cell mRNA profile that mostly were not accompanied by similar trends in proteomic profile. Observed elimination of transcript pool from translation process may occur due to the reduced stability of initiating complexes for some transcripts. Earlier, we demonstrated that sequences of ribosomal binding sites and other determinants of 5‘-UTR together with mRNA abundance can be accounted for up to 72% of protein level variations under exponential growth in *M*. *gallisepticum* ^11^. However, when analyzing the response to heat shock, prognostic value of this model dramatically decreases. One of the possible explanations for this phenomenon is the rearrangement of ribosome complex. Heterogeneity of ribosomal protein composition was described more than 40 years ago^23–25^. Later, an idea of “ribosomal filter” was introduced for eukaryotes. This filter acts due to the variable affinity of mRNA to various ribosomal proteins^19^. Thus, the term “ribosomal code” was introduced^25^. We demonstrated changes in the abundance of 11 ribosomal proteins and translation factors (Efp, FusA, InfC, PrfA, RplA, RplW, RpmC, RpmF, RpmG, RpsH, and RpsM) (Supplementary Table S1), i.e., changes in the stoichiometry of ribosome-related proteins can be hypothesized. Although the functions of most ribosomal proteins are not yet described, most of them are known to stabilize ribosomal subunits and guide their folding. Some researchers suggested possible moonlighting for several ribosomal proteins^3^. Hence, the arrangement of protein component of translational complex during heat shock response can provide both the specificity of proteomic profile changes and the stability of ribosomal subunit. Aminoacyl-tRNA modifications are another possible regulatory mechanism. We observed increased abundance of five aa-tRNA-synthetases (AspS, GlyS, IleS, ProS, SerS) and two enzymes (TilS, MnmG/GidA), which modify nucleotide in wobble position of some aa–tRNAs (Supplementary Table S1).

While dynamic changes in antigen repertoire in Mycoplasmas during infection is a well-described phenomenon, studied on transcriptional^26^ and protein^6^ levels, we observe here overall reduction of hemagglutination potential induced by heat shock which is accompanied by drop in abundance of several major antigens. This discrepancy might in fact reflect difference in the nature of models used – phase transition was observed in models where host-pathogen interaction occurs directly, while our current model is focused on a single acute triggering factor – fever, represented here by heat shock *in vitro*.

When analyzing proteome profiling data, we revealed increased NADH-oxidase (GCW_02755) levels (Supplementary Table S1). This protein catalyzes NADH oxidation to NAD^+^ involving oxygen with water molecule acting as electron acceptor. This reaction generates peroxide, the potential source of reactive oxygen species which are the key endogenous mutagens. Therefore, we have also observed an increase in the representation of proteins such as OsmC, PotA, and TrxA_2 involved in protecting cells from oxidative stress.

Published data suggest that cells require more ATP under heat shock; ATP is actively utilized by chaperones to restore regular folding of cell proteins^27^. Significant accumulation of ATP by *M*. *gallisepticum* cells (ATP concentration rises 7-fold or more) is of special interest (see Fig. 7), because even *E*. *coli* demonstrates just twofold increase in ATP levels because of UV-induced SOS-response^28^. In eukaryotic cells, ATP levels reduce by 50% under heat stress^29^. The study of ATP consumption and synthesis in *M*. *pneumoniae* revealed that mycoplasma metabolism does not promote or inhibit the production of macroergic compounds to force synthetic or catabolic processes when culturing *M*. *pneumoniae* in a synthetic medium without stresses. It was shown that glycolysis is the only source of ATP in *M*. *pneumoniae* and enzymes of this path are the most represented in the total proteome thus compensating energy deficiency^3^. However, our data suggests that *M*. *gallisepticum* can change cell energy potential and adapt it to subsequent reactions during the heat stress without affecting catabolism or synthesis greatly.

Considering that glycolysis is the only source of ATP in *M*. *gallisepticum* and NAD^+^ regeneration is the limiting stage of glycolysis, we hypothesized that increased intracellular NADH-oxidase is required to accelerate glycolysis and ATP synthesis. Moreover, the conversion of pyruvate into acetate instead of lactate provides cell with an additional ATP molecule per pyruvate molecule^6^. Therefore, the phenomenon described above presumably is another evidence on the fact many mycoplasmas generate reactive oxygen species in response to stresses^30–32^. This is a common mechanism that enables Mollicute pathogenicity. It should be noted that it is triggered by rapid exposure of the cell culture to increased temperature which occurs as a part of host’s immune response.

Increased levels of free nitrogenous bases and nucleosides as compared with nucleotides conform to changes in cell energy state. In addition, increased NAD and reduced NAD synthesis metabolites, nicotinamide and nicotinamide nucleotide, also indicate changes in energy state (see Supplementary Table S3).

Also, we detected changes in terpenoid biosynthesis metabolite levels in cells exposed to heat shock (see Figs 6 and S1). Terpenoids are bioorganic compounds whose carbon skeleton is composed of isoprene chains. They are vital for many microorganisms^33^. The virulence of numerous pathogenic bacteria is realized via MEP/DOXP pathway of terpenoid biosynthesis (mycoplasmas have retained this exact pathway during the reductive evolution). Isoprenoids, terpenoids synthesized from isopentenyl phosphate, are required to infect and to persist in hosts by a wide range of pathogens, for example, *M*. *paratuberculosis*, *K*. *pneumoniae*, *S*. *typhimurium*, *H*. *pylori*, and *C*. *trachomatis* ^33–37^. Thus, the ability of *M*. *paratuberculosis gcpE* transposon mutant to colonize liver and intestine of BALB/c mice decreases after perinatal infection^36^. Lai *et al*.^35^ demonstrated that *dxr* (gene encoding 1-deoxy-D-xylulose-5-phosphate reductoisomerase, an enzyme of MEP/DOXP terpenoid biosynthesis pathway) is actively expressed in *K*. *pneumoniae* under perinatal infection of BALB/c mice. Another component of terpenoid biosynthesis, *dxs*, is activated in *B*. *abortus* that survives in macrophages^37^. It was also shown that 1-hydroxy-2-methyl-2-(E)-butenyl 4-diphosphate, intermediate metabolite of terpenoid biosynthesis pathway, can activate human Vγ9/Vδ2 T cells^33^. We suppose that since terpenoid biosynthesis pathway is affected by heat stress in mycoplasmas as well, it may play its role during infection processes. Despite general reduction of metabolic pathways in *M*. *gallisepticum*, this pathway was previously described in detail^14^. Currently, the role of terpenoid biosynthesis pathway in *M*. *gallisepticum* is still unclear. However, in our previous work we revealed the role of its enzymes in *M*. *gallisepticum* adaptation to various exposures using non-targeted mutagenesis of genes responsible for terpenoid synthesis in *M*. *gallisepticum* ^38^. Mutant *M*. *gallisepticum* demonstrated modified colonial morphology and significantly decreased growth as compared with the wild-type strain. In addition, our experiments with mutant mycoplasma cells indicate that terpenoids are actively involved in protection against reactive oxygen species generated during thermal adaptation. It is not typical for this pathogen and can be considered as an enzymatic machinery that protects Mollicutes from reactive oxygen species generated during the process of infection. Direct involvement of terpenoid synthesis into the protection from reactive oxygen species was previously demonstrated for *B*. *subtilis* only. Cyclization of linear terpenoids in *B*. *subtilis* spores triggered by reactive oxygen species provides protective effect *in vivo* ^39^. Phylogenetic analysis indicates that *B*. *subtilis* is considered to be the closest evolutionary precursor of mycoplasma; similar mechanism was probably inherited from this microbe.

L-ornithine and L-citrulline, the components of ADI pathway, are among metabolites whose levels significantly reduce under heat stress (see Supplementary Fig. 1). The main step of ADI pathway is arginine conversion to citrulline; this reaction is catalyzed by arginine deiminase (ArcA) and produces ammonia^40^. ArcA in *M*. *gallisepticum* probably functions in the reverse direction thus synthesizing arginine; thus, decreased citrulline can be accounted for by increased amino acid requirements for protein translation while ornithine is the product of citrulline degradation.

In summary, we have performed accurate proteome-wide characterization of *M*. *gallisepticum* adaptation to elevated temperature. Besides the increase in abundance of heat stress-related proteins (i.e., chaperones and several classes of proteins involved in translation process), most changes in *M*. *gallisepticum* proteome under heat stress are either low or insignificant despite genome-wide and intensive response at transcription level. The observed discrepancy is supposed to be connected with major rearrangement in ribosome-related proteins repertoire and, possibly, reconfiguration of ribosome stoichiometry. As mycoplasmas regulatory and metabolic potentials are significantly reduced, alternative adaptation strategy can be proposed – *M*. *gallisepticum* tends to maintain its proteome stable rather than balanced or optimal despite wide and intense response at transcription level. This kind of adaptation requires minimal regulatory systems and promotes accumulation of ATP in amounts unexpected for *M*. *gallisepticum*. The latter and several other components of complex proteome and metabolome level response (drop of hemagglutination and reduction in major antigens’ abundance, changes in terpenoid biosynthesis pathway) can’t be directly attributed to heat shock response, but might be relevant to mycoplasma’s pathogenic potential. In that case, fever during host’s immune response may serve as a trigger for rapid development of acute infection, evasion of immune system and dissemination of pathogen.

## Materials and Methods

### Cell cultures

*Mycoplasma gallisepticum* S6 was cultivated at 37 °C on a modified Edwards medium containing tryptose (20 g/L), Tris (3 g/L), NaCl (5 g/L), KCl (5 g/L), yeast dialysate (5%), horse serum (10%) and glucose (1%) at pH 7.4 in aerobic conditions for 12 hours before heat chock, which corresponds to exponential phase. The cells were exposed to sublethal heat shock, i.e. cells were cultivated at 46 °C for 30 minutes and then conditioned for 2 hours at 37 °C and compared to initial culture sampled right before inducing heat shock. All conditions mentioned above were justified previously^12^. Cells were harvested by centrifugation at 8000 rcf and 4 °C for 10 minutes.

### Tryptic digestion in solution

Protein extraction and trypsin digestion were performed as described previously^6^.

### Protein identification

To generate protein list and ion library for further quantitative analysis 6 information-dependent acquisition runs were performed on Sciex TripleTOF 5600+ QTOF mass-spectrometer coupled to Eksigent NanoLC Ultra 2D+ nano-HPLC system through Sciex NanoSpray III nano-ESI ion source. The gradient length was 2 hours. Identification was performed with Sciex ProteinPilot 4.5 software against a database of all proteins of *M*. *gallisepticum* strain S6 (GenBank ID: AFFR01000000). A cut-off of 1% global FDR at protein level and a minimum of 2 identified peptides was applied. To estimate absolute concentration a search with Andromeda engine (as embedded in MaxQuant 1.5.5 software) was performed and obtained iBAQ values were used to predict copy number per cell with linear regression using estimates made for a closely related species *Mycoplasma pneumoniae* ^3^.Detailed procedures can be found in Supplementary Information.

### Large-scale protein quantification with SWATH acquisition

Large-scale quantitative LC-MS analysis was performed on 6 independent biological replicates utilizing DIA SWATH technology. Raw data was obtained by triplicate injection of each sample with LC parameters and configuration identical to IDA experiments.

To process SWATH data, protein identification lists (group files) obtained for IDA experiments were translated into ion library with PeakView 2.0 (Sciex) SWATH processing tools. The ion library was built in a way that ensured that all observed peptides with all identified fragments to be used for signal extraction. Extraction window was set to 15 min, mass window was set to 50 ppm. The next step as suggested by the manufacturer (Sciex) proposes direct comparison of peptide intensities (as sum of fragment intensities) per protein, however this way almost completely lacks quality control. To enhance reliability, we filtered extracted ion chromatogram data starting from ion level (that is for each pair of parent and fragment ion in the ion library separately) for reliably quantifiable proteins with the use of a homemade script in R. The script is described in detail in Supplementary Information. In brief, it included several steps of scaling normalization and averaging of replicates at LC-MS acquisition, sample preparation and biological levels and selection of “best flyer”^41^ peptides and reproducible and interference-free transitions. Top 3 most abundant peptides with not less than 3 qualified transitions were used to produce protein intensity (as sum intensities of selected ions) in every biological sample. Difference between control, temperature exposed and further incubated cultures and its significance on biological level was evaluated for each qualified protein on log-transformed intensities with Student’s t-test.

### Metabolomic analysis

For metabolite analysis *M*. *gallisepticum* were grown in liquid medium. The metabolism of the growing culture was rapidly quenched by cold methanol extraction^42^. Mass-spectrometry analysis was performed on a Q-TOF 6520 series time-of-flight mass spectrometer (Agilent Technologies). The flow from the analytical column was introduced directly into the electrospray ion source of the mass spectrometer. The metabolite search and data processing were performed using Metabolomic Analysis and Visualization Engine (MAVEN)^43^ software and the Internet resource called the Trans-Proteomic Pipeline (TPP), where the data were converted into the mzXML format (MAVEN compatible).A pairwise sample comparison of metabolomic data for *M*. *gallisepticum* in normal and heat conditions was performed using the XCMS online service, providing a direct comparison of two sample groups. From the resulting features, we selected only those with a p value ≤ 0.05 in order to state that compound concentration is different in two conditions. All metabolic experiments were made in three biological and two technical replicates. Detailed procedure can be found in Supplementary Information.

### Targeted MRM

Validation of massive quantification by SWATH was performed with MRM methodology on QTRAP 4500 (Sciex, USA) triple quadrupole mass spectrometer equipped with a NanoSpray III ion source (Sciex) coupled to an expert NanoLC 400 nano-HPLC system (Eksigent, USA). Validation was performed with 3 independent biological replicates. Protein list was composed from proteins exhibiting different scale of response as detected in large-scale quantitation effort and a list of additional proteins that were not quantified successfully. Detailed procedures were described previously^6^.

### Two-dimensional difference gel electrophoresis (2DE)

The 2DE, samples trypsin digestion with subsequent MALDI analysis were performed as previously described^44^.

### Hemagglutination assay

For hemagglutination assay glutaraldehyde-stabilized chicken red blood cells (RBCs, Sigma-Aldrich) were used. Briefly, RBCs were resuspended in PBS according to manufacturer protocol, mixed with sodium citrate buffer (1:3, respectively) and centrifuged at 500 rpm for 5 min. Upper phase was removed, RBCs were resuspended in PBS and centrifuged at 500 rpm for 5 min. Procedure was repeated 2 times. Then series of two-fold dilutions of *M*. *gallisepticum* culture in PBS were incubated with an aliquot of RBCs until reaction was developed.

### Measurement of ATP concentration in M. gallisepticum cells

Detection of ATP was performed as previously described^6^.

## Electronic supplementary material

Supplementary Information

Table S1

Table S2

Table S3
