# Supplementary material for: Response induced in Mycoplasma gallisepticum under heat shock might be relevant to infection process
Source: Sci Rep. 2017 Sep 12;7:11330. doi: 10.1038/s41598-017-09237-7 (PMC5595898; doi:10.1038/s41598-017-09237-7)
Supplement: Supplementary file 1 — Supplementary Information [file 41598_2017_9237_MOESM1_ESM.pdf]

Response induced in *Mycoplasma gallisepticum* under heat shock might be relevant to infection process.

Ivan Butenko[1], Anna Vanyushkina[1], Olga Pobeguts[1], Daria Matyushkina[1*], Sergey Kovalchuk[1,2], Alexey Gorbachev[1], Nicolay Anikanov[2], Gleb Fisunov[1], Vadim Govorun[1,2,3]

1 Laboratory of Proteomic Analysis, Federal Research and Clinical Centre of Physical-Chemical Medicine, Moscow 119435, Russia

2 Laboratory of Proteomics, Shemyakin-Ovchinnikov Institute of Bioorganic Chemistry, Moscow 117997, Russia

3 Moscow Institute of Physics and Technology (State University), Dolgoprudny 141700, Russia

***d.matyushkina@gmail.com**

Supplementary methods

Protein identification

Analysis was performed on a TripleTOF 5600+ mass-spectrometer with a NanoSpray III ion source (both Sciex, USA) coupled to a NanoLC Ultra 2D+ nano-HPLC system (Eksigent, USA). The HPLC system was configured in a trap-elute mode. For a sample

loading solvent and solvent A, the mix of 98.9% water, 1% methanol, 0.1% formic acid (v/v) was used. Solvent B was 99.9% acetonitrile, 0.1% formic acid (v/v). Samples were loaded on a trap column (ChromXP C18, 3 μm, 120 Å 350 μm × 0.5 mm, Eksigent) at a flow rate of 3 μl/min over 10 min and eluted through the separation column (3C18-CL-120, 3 μm, 120 Å 75 μm × 150 mm, Eksigent) at a flow rate of 300 nl/min. The gradient was from 5 to 40% of solvent B in 120 min. The column and the precolumn were regenerated between runs by washing with 95% of solvent B for 7 min and equilibrated with 5% of solvent B for 25 min. Between the samples to ensure the absence of carryover both the column and the precolumn were thoroughly washed with a blank injection trap-elute gradient that included five 7 minute 5-95-95-5%B waves followed by 25 min 5%B equilibration.

Mass spectra were acquired in a positive ion mode. Information-dependent mass-spectrometer experiment included 1 survey scan followed by 50 dependent fragmentation scans. Survey spectra acquisition parameters were as follows: mass range for analysis and subsequent ion selection for fragmentation was 300-1250 m/z, signal accumulation time was 250 ms. Ions for fragmentation were selected based on intensity with the threshold of 200 cps and the charge state from 2 to 5. Fragmentation spectra acquisition parameters were as follows: resolution of quadrupole was set to Unit (0.7 Da), measurement mass range was 200-1800 m/z, optimization of ion beam focus was to obtain maximal sensitivity, signal accumulation time was 50 ms for each parent ion. Collision activated dissociation was performed with nitrogen gas with collision energy ramping from 25 to 55 V within 50 ms signal accumulation time. Analyzed parent ions were sent to dynamic exclusion list for 15 s in order to get the next fragmentation spectra of the same compound around its chromatographic peak apex (minimum peak width throughout the gradient was about 30 s).

For protein identification, .wiff data files were analyzed with ProteinPilot 4.5 revision 1656 (Sciex) using search algorithm Paragon 4.5.0.0 revision 1654 (Sciex) and a standard set of identification settings to search against a database of all proteins of *M. gallisepticum* strain S6 (GenBank ID: AFFR01000000). The following parameters were used: alkylation of cysteine - iodoacetamide, trypsin digestion, TripleTOF 5600 equipment, species: none, thorough search with additional statistical FDR analysis. Peptide identifications were processed with default settings by a ProteinPilot software built-in ProGroup algorithm. The final protein identification list was obtained with the threshold reliable protein ID unused score calculated by ProteomicS Performance Evaluation Pipeline Software (PSPEP, Sciex) algorithm for 1% global FDR from fit.

Large-scale protein quantification with SWATH acquisition

Large-scale quantitative LC-MS analysis was performed on 6 independent biological replicates utilizing DIA SWATH technology. Raw data was obtained by triplicate injection of each sample with identical to IDA experiments LC parameters and configuration. The acquisition parameters were as follows: full ~3.3 sec cycle included one 50 ms survey scan for m/z 300-1250, followed by 32 SWATH windows 20 Da each spanning 400-1000 m/z range. SWATH data was obtained in high sensitivity mode with 100 ms accumulation time for each spectrum.

To process SWATH data, protein identification lists (group files) obtained for IDA experiments were translated into ion library with PeakView 2.0 (Sciex) SWATH processing tools for the number of proteins falling into 1% global FDR from fit for the corresponding group file and reported peptide confidence equal to 99 with the exclusion of shared and modified peptides (except for carbamylation of cysteines). The ion library was used to obtain

extracted ion chromatograms for corresponding transitions from SWATH data files with the following parameters: 2000 peptides per protein and 2000 fragments per peptide (that is to extract all observed peptides with all identified fragments), extraction window 15 min, mass window 50 ppm. The next step as suggested by the manufacturer (Sciex) proposes direct comparison of summary peptide intensities (as summary fragment intensities) per protein, however this way almost completely lacks of quality control. To enhance reliability, we filtered extracted ion chromatogram data starting from ion level (that is for each pair of parent and fragment ion in the ion library separately) for reliably quantifiable proteins with the use of a homemade script in R. The algorithm included scaling normalization and averaging of three technical repeats per sample (each sample was analyzed in triplicate;thus, this step allows for injection and LC-MS signal reproducibility), exclusion of peptides with less than 3 quantified fragments and exclusion of protein with less than 3 quantified peptides, followed by separation of quantifiable transitions. The latter was based on the assumption of the proportionality of fragment ion intensity to parent ion intensity and, consequently, the proportionality of fragment intensity changes between different samples 1. Quantifiable fragment ions were selected based on trend search within all the fragments on the basis of normalized spectral contrast angle[1] analysis with iterative search of fragment clusters. Minimal number of fragments that must follow the same trend (i.e. follow reproducible fragmentation pattern) between samples to be used as “reliable” was selected to be 3. After fragment filtering all proteins with less than 3 peptides were excluded. This was followed by renormalization of different LC-MS repeats of same sample and averaging, then by the second normalization and averaging step between technical repeats of trypsinolysis of the same sample (some biological samples were trypsinized in replicates) and the third normalization and averaging step within sample type. To obtain results biological samples

were cross scale-normalized based on the assumption that most of the cell proteins in any pair of samples were supposed to be independent (that is the scaling should set the average difference between proteins for a pair of samples to minimum).

To calculate protein fold change results, for all fragment intensities logarithm with the base B (was chosen to obtain the best scaling) was taken. The logarithm results for each fragment were averaged within a sample (LC-MS repeats*trypsinolysis repeats*technical replicates) to obtain peptide logarithm result. Protein result was calculated as a median of its 3 “best flyer”[2] (ones with largest signal) peptide values. Fold change for a protein was calculated as a difference in median values between samples raised to power B.

Metabolomic analysis

The following chemicals were used as standards: sodium pyruvate (100 mg/ml, disodium salt hydrate), D-fructose 6-phosphate, hydrate of sodium phosphoenolpyruvate (97% purity, enzyme quality), dehydrated disodium salt of D-ribose 5-phosphate, di-glyceraldehyde 3-phosphate (46.1 mg/ml). Purified (98%) amino acids, nucleotides, nucleosides (adenosine, deoxyadenosine, inosine, cytosine monophosphate, and thymidine) from Sigma-Aldrich (USA) were used as standards as well. The following samples were used for extraction and solution preparation: absolute methanol (HPLC grade) from Biosolve (The Netherlands), ammonium acetate (ultra clean grade) from Helicon (Russia), formic acid (98-100%) from Riedel-de Haen (Germany), ammonium hydroxide solution (29.73%) from Fisher Scientific (USA), water (HPLC-MS) and acetonitrile (HPLC-MS) from Panreac (Spain). The following chromatographic analytical columns were used in the study: Luna NH2 (30 mm × 2 mm × 5 µm) from Phenomenex Torrance (USA) and Zorbax RX-SIL Narrow-Bore (150 mm × 2.1 mm × 5 µm) from Agilent Technologies (USA). To protect the

separating phase of these highly effective analytical columns from chemical damage, the following safety cartridges were used: Zorbax NH2 4-Pack analytical guard column (4.6 mm × 12.5 mm × 5 μm) and Zorbax RX-SIL 4-Pack analytical guard column (4.6 mm × 12.5 mm × 5 μm) purchased from Agilent Technologies.

For metabolite analysis *M. gallisepticum* were grown in liquid medium. The metabolism of the growing culture was rapidly quenched by cold methanol extraction. A cold methanol extraction method was developed on the basis of a previously reported cold methanol extraction protocol[3] as described below.

Bacterial suspensions (50 ml) were centrifuged for 15 min at 16,000g at 4°C, and the supernatants were discarded. The precipitate was resuspended in 3 ml of 150 mM NaCl, and the resulting suspension was centrifuged for 10 min at 16,000g at 4°C. The supernatant was discarded. The precipitate was resuspended in 150 μl of 150 mM NaCl and centrifuged for 10 min at 16,000g at 4°C. The supernatant was discarded. The washing was repeated one more time. The precipitate was resuspended in 75 μl of 150 mM NaCl. The metabolites were extracted by adding of 1000 μl of methanol (–77°C) to the sample; the sample was vigorously shaken (1 min) and then kept at –77°C for 15 min. The sample was warmed for 3 min and then thoroughly shaken again. The resulting sample was centrifuged for 30 min at 16,000g at 4°C. The supernatant was separated into aliquots and lyophilized. The dry extract was kept no longer than 4 days prior to analysis. The dry extract was dissolved in mixture consisting of 20% acetonitrile and 80% water and analyzed immediately.

Mass-spectrometry analysis was performed on a Q-TOF 6520 series time-of-flight mass spectrometer (Agilent Technologies). The flow from the analytical column was introduced directly into the electrospray ion source of the mass spectrometer. Prior to the experiment, the mass spectrometer was calibrated to 2 ppm accuracy of the m/z value. The

ionizing spray voltage was 3500 V in both positive and negative, ionization modes. Nitrogen of various degrees of purity was used as a dry gas at a pressure of 20 psi with flow rate 6 l/min and as a gas in the collision cell. The temperature of the quartz capillary was 325°C. HPLC-MS and HPLC-MS/MS analysis was carried out using the series 1200 high performance liquid chromatograph (Agilent Technologies) coupled with the mass spectrometer. Chromatographic analysis was performed with the following parameters: auto-sampling temperature, 8°C; analytical column temperature, 18°C; injection volume, 2 µl; solvent flow rate, 50 µl/min. The following solvents were used as eluting solutions: eluent A was 20 mM ammonium acetate / 0.25 mM ammonium hydroxide in water/acetonitrile mixture of 95:5 ratio, pH 8.02; eluent B was pure acetonitrile. The gradient of the solvent transition was as follows: for positive ionization mode t = 0, 100% B; t = 30 min, 0% B; t = 32 min, 0% B; t = 35 min, 100% B; t = 60 min, 100% B. In negative ionization mode t = 0, 100% B; t = 30 min, 0% B; t = 32 min, 0% B; t = 35 min, 100% B; t = 60 min, 100% B. Metabolite identification was confirmed by fragmentation spectra of the detected ions. The collision energy was fixed at 20 eV, and MS/MS spectra were recorded in the range of 30-1000 m/z with the minimal width of the ion isolation window at 1.3 m/z.

The metabolite search and data processing were performed using Metabolomic Analysis and Visualization Engine (MAVEN)[4] software and the Internet resource called the Trans-Proteomic Pipeline (TPP), where the data were converted into the mzXML format (MAVEN compatible). *M. gallisepticum* metabolites were identified using the list of all possible metabolites for these bacteria, garnered from all the protein annotation data previously reported by our lab[5]. A list of all theoretically possible metabolites of *M. gallisepticum* was prepared in accordance with the KEGG database as described previously[6] and contained all the metabolites associated with the proteins that were annotated for this

bacteria. The following parameters were used for the search: range of m/z values (extraction window), 15 ppm m/z; minimum intensity of peak, 1000 a.u.; minimum value of baseline signal intensity ratio, 10.

A pairwise sample comparison of metabolomic data for *M. gallisepticum* in normal and heat conditions was performed using the XCMS online service, providing a direct comparison of two sample groups. From the resulting features, we selected only those with a p value ≤ 0.05 in order to state that compound concentration is different in two conditions. Additionally, these compounds were analyzed manually by counting the metabolite identifications among the repetitions of normal and stressed conditions for both acquisition modes. The quantification of an identified metabolite is possible only if it was detected at least three times in both cases. In the case a compound was detected less than three times in all repetitions for same grow condition, only a qualitative comparison is possible, based on the detection of MS counts for this compound in the group.

References

1. Toprak, U. H. *et al.* Conserved peptide fragmentation as a benchmarking tool for mass spectrometers and a discriminating feature for targeted proteomics. *Mol. Cell. Proteomics* **13,** 2056–71 (2014).

2. Ludwig, C., Claassen, M., Schmidt, A. & Aebersold, R. Estimation of Absolute Protein Quantities of Unlabeled Samples by Selected Reaction Monitoring Mass Spectrometry. *Mol. Cell. Proteomics* **11,** M111.013987-M111.013987 (2012).

3. Maharjan, R. P. & Ferenci, T. Global metabolite analysis: the influence of extraction methodology on metabolome profiles of Escherichia coli. *Anal. Biochem.***313,** 145–54 (2003).

4. Melamud, E., Vastag, L. & Rabinowitz, J. D. Metabolomic Analysis and Visualization Engine for LC−MS Data. *Anal. Chem.***82,** 9818–9826 (2010).

5. Fisunov, G. *et al.* Core Proteome of the Minimal Cell: Comparative Proteomics of Three Mollicute Species. *PLoS One* **6,** e21964 (2011).

6. Vanyushkina, A., Fisunov, G., Gorbachev, A. Y., Kamashev, D. E. & Govorun, V. Metabolomic analysis of three Mollicute species. *PLoS One* **9,** e89312 (2014).

Supplementary figures

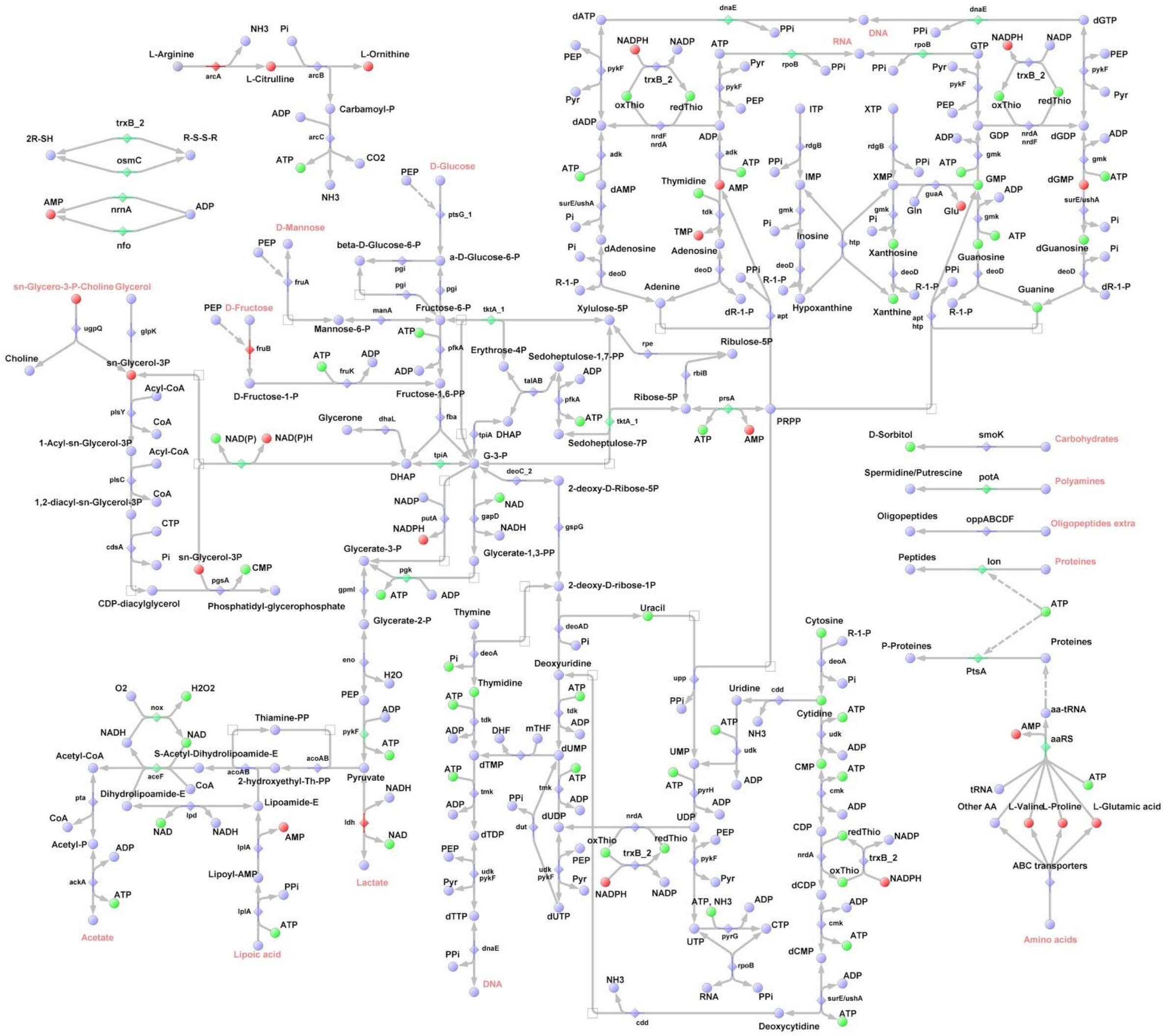

S1 Figure. Reconstructed metabolic map of *M. gallisepticum S6* under heat stress.

Biochemical reactions of terpenoids biosynthesis annotated for *M. gallisepticum S6* indicated in violet. Metabolites involved in this pathway are shown as circles; enzymes are shown as diamonds; Metabolites and proteins that demonstrated increased level under heat shock marked in green, decreased level – in red.
